# An increase of cereal intake as an approach to weight reduction in children is effective only when accompanied by nutrition education: a randomized controlled trial

**DOI:** 10.1186/1475-2891-7-28

**Published:** 2008-09-10

**Authors:** Jorge L Rosado, María del R Arellano, Karina Montemayor, Olga P García, María del C Caamaño

**Affiliations:** 1 Facultad de Ciencias Naturales, Universidad Autónoma de Querétaro, Querétaro México; 2 CINDETEC, México

## Abstract

**Background:**

The main emphasis of dietary advice for control of obesity has been on reducing dietary fat. Increasing ready to eat cereal (RTEC) consumption could be a strategy to reduce fat intake and increase carbohydrate intake resulting in a diet with lower energy density.

**Objectives:**

1. To determine if an increase in RTEC intake is an effective strategy to reduce excess body weight and blood lipids in overweight or at risk of overweight children. 2. To determine if a nutrition education program would make a difference on the response to an increase in cereal intake. 3) To determine if increase in RTEC intake alone or with a nutrition education program has an effect on plasma lipid profile.

**Experimental design:**

One hundred and forty seven overweight or at risk of overweight children (6–12 y of age) were assigned to one of four different treatments: a. One serving of 33 ± 7 g of RTEC for breakfast; b. one serving of 33 ± 7 g of RTEC for breakfast and another one for dinner; c. one serving of 33 ± 7 g of RTEC for breakfast and a nutrition education program. d. Non intervention, control group. Anthropometry, body composition, physical activity and blood lipids were measured at baseline, before treatments, and 12 weeks after treatments.

**Results:**

After 12 weeks of intervention only the children that received 33 ± 7 g of RTEC and nutrition education had significantly lower body weight [-1.01 (-1.69, -0.34) ], p < 0.01], lower BMI [-0.95 (-1.71, -0.20), p < 0.01] and lower total body fat [-0.71 (-1.71, 0.28), p < 0.05] compared with the control group [1.19 (0.39, 1.98), 0.01 (-0.38, 0.41), 0.44 (-0.46, 1.35) respectively]. Plasma triglycerides and VLDL were significantly reduced [-20.74 (-36.44, -5.05), -3.78 (-6.91, -0.64) respectively, p < 0.05] and HDL increased significantly [6.61 (2.15, 11.08), p < 0.01] only in this treatment group. The groups that received 1 or 2 doses of RTEC alone were not significantly different to the control group.

**Conclusion:**

A strategy to increase RTEC consumption, as a source of carbohydrate, to reduce obesity is effective only when accompanied by nutrition education. The need for education could be extrapolated to other strategies intended for treatment of obesity.

**Trial Registration:**

Australian New Zealand Clincial Trial Registry. Request no: ACTRN12608000025336

## Background

Recent estimates suggest that up to 1.7 billion people worldwide are overweight or obese, making it one of the biggest health threats facing world's population. Obesity lies at the other end of malnutrition scale and is becoming a public health problem in developing countries as well. Over 115 million people suffer from obesity related problems in developing countries [1]. In Brazil and Colombia for example, 36 and 41% of the population respectively is overweight. Prevalence of obesity in Mexico was unknown until recently [2,3]: about 26% of children between 5 and 12 years of age and 35% of the adult women are obese. The high prevalence of obesity in the Mexican population must be contributing to the increment in chronic diseases that has been observed in recent years [4]. Health officials and academia have recognized the need for urgent preventive measures to stop this accelerating trend.

Several studies have identified an excessive intake of dietary fat as a major mechanism for increasing the amount of body fat in humans and experimental animals. Diets with a high fat content are energy dense [5]. Thus, reduction of dietary fat as a treatment for obesity has been a widely used approach. A number of trials with low-fat diets have demonstrated the effectiveness of such recommendation [6-8]. In addition to weight loss, low fat diets help maintain low cholesterol and triglyceride levels in blood, reduce leptin concentration, increase adiponectin and reduce insulin resistance, and decrease cardiovascular and diabetes risk [9,10].

An increase in the carbohydrate to fat ratio is associated with the reduction in energy density of the diet [11]. A dietary recommendation to increase cereal consumption is a possible approach to improve the carbohydrate to fat ratio. Studies in adult men and women have demonstrated that an increase in dietary carbohydrates from ready-to-eat cereals (RTEC) or other foods, even in the lack of an advice to reduce fat, is a potentially effective approach for weight reduction [5,12,13].

The objectives of the study were: 1) To determine if an increase in cereal intake by consuming RTEC, among overweight or at risk of overweight children is an effective treatment to reduce excess body fat, 2) To determine if the inclusion of a nutrition education program in addition to an increase in carbohydrate intake has an effect on body weight and body fat, and 3) To determine if an increase in RTEC intake alone or with a nutrition education program has an effect on plasma lipid profile.

## Methods

### Subjects and place of study

Children were eligible if they had a BMI for age > 85% and were attending elementary school with an age range from 6 to 12 years. In order to detect children as being overweight or at risk of overweight, 6 elementary schools of the city of Queretaro were randomly selected and invited to participate; 5 schools accepted participation. Parents of all children from 1^st ^to 6^th ^grade were invited to a session where details of the study were explained, including benefits and potential risks of child participation. Parents of 905 children accepted voluntarily to participate in an initial screening to detect overweight or at risk of overweight children. Weight and height were determined in all children at their schools. Children were weighed without sweater or jacket and without shoes using an electronic scale (SECA, Erecta 844, Hamburg, Germany) to the nearest 1 g. Height was measured using portable stadimeters (SECA, Bodymeter 208, Germany). Children with a BMI-for-age above the 85^th ^percentile were enrolled in the experimental study. According to the Center for Disease Control and Prevention (CDC) references, a child at risk of overweight is defined as having a BMI-for-age between the 85^th ^and 95^th ^percentile of the CDC growth charts [14]. Overweight is defined as a BMI-for age at or above the 95^th ^percentile (14).

Of the 905 children initially screened, 17% had a BMI-for-age percentile between 85% and 95%, and 18% had a BMI-for-age percentile equal or above 95%. Of these overweight and at risk of overweight children, 256 accepted to participate in a longitudinal controlled study, from which 178 children completed the study. Lost to follow-up was mainly due to the children's lack of compliance to the study protocol. The sample size of 178 subjects that completed the study accomplishes the expected sample size with an alpha error of 0.05 and a beta error of 0.2, to detect a BMI expected difference of 1 kg/m^2^, with an expected standard deviation of BMI change of 1 kg/m^2^. Blood samples were taken from children if parents agreed to the procedure. Of the 178 subjects that completed the study, a blood sample was obtained from 129 children. Children included in the study were healthy volunteers with no apparent disease apart from being overweight.

### Experimental groups and treatments

Children were randomly assigned to one of four different treatments. They were stratified into 4 groups with similar age, height and BMI percentile and same gender, in order to create groups with similar baseline characteristics. A randomization of treatments was done to each group with a computer generating random number list. The randomization was done at a central office by someone who did not have contact with the children or their parents. Children in group 1 consumed one serving of 33 ± 7 g of RTEC (Kellogg's de Mexico, Querétaro, Mexico) at breakfast. Children in group 2 consumed two servings of 33 ± 7 g of RTEC, one at breakfast and another serving at dinner. Children in group 3 consumed one serving of 33 ± 7 grams of RTEC and in addition, both children and mothers received a nutrition education guide that contained recommendations for healthy eating. Children in group 4 were involved in the study and had no treatment. Follow up in all groups was for 12 weeks.

To allow for variety in the diet, children consumed from 4 different types of RTEC: a corn based RTEC (Corn Flakes^®^, Kellogg Company Mexico), a pre-sweetened corn based RTEC (Zucaritas^®^, Kellogg Company Mexico), a pre-sweetened corn based, chocolate flavored RTEC (Choco Zucaritas^®^, Kellogg Company Mexico), and a pre-sweetened rice based, chocolate flavored RTEC (ChocoKrispis^®^, Kellogg Company Mexico). These RTEC were chosen because of the high consumption among children. The children were allowed to choose from the 3 pre-sweetened RTEC only for 3 days in one week and were not allowed to repeat. The remaining four days children consumed from corn-based cereal only. The mean nutrient composition of RTEC per serving was as follows: 165 Kcal (712 KJ), 5.8 g of protein, 0.5 g total fat, and 35 g of carbohydrates. The RTEC was consumed with 250 mL of cold skimmed milk in a bowl with a spoon. Compliance was recorded by weekly interviews to the parents.

A nutrition education guide was prepared by one of us (RA) based on general recommendations for obese individual developed by Perez-Lizaur and Marvan [15] which included recommendations for the whole family and recommendations for the child. The nutrition education program included 12 sessions (one per week) that were given at school to the children's parents (usually the mother), both in oral and written form. The dietary recommendations were given by a nutritionist. Practice of the recommendations mentioned above was monitored weekly during RTEC delivery at the school by asking the parents if they had any difficulty following the nutrition education guide. Table 1 shows a summary of the major aspects included in the nutrition education guide. As part of the education guide, a sample menu was provided so that parents could use it to plan their meals at home and for school.

**Table 1:** Summary of the nutrition education guide used in one treatment group

Recommendations concerning the family:
• Parents are responsible for teaching their children healthy food choices in and outside their home.
• Lunch must be a simple, appetizing, easy to carry, economical and nutritious meal.
• When eating together, the rest of the family should eat the same type of foods, but the serving size may vary individually.
• Mealtimes at home should be calmed and trouble-free; this is not a good time for arguments about the child's diet.
• The amount of foods eaten at home is influenced by family preferences. Watch out what is bought and stored at home. Avoid storing foods that the child may crave for such as deserts, soft drinks, candies, potato chips and other calorie dense foods.
Recommendations concerning the child
• The child must continue with his/her usual physical activity.
• Food preferences should be considered when planning the child's meals.
• A child must always have breakfast before school, or during the weekend.
• Consume only skimmed milk, low-fat cheese and low-fat yogurt.
• Eat the regular foods prepared at home, following the general recommendations given in the sample menu.
• If there is a mealtime out of home, child may be allowed to eat the foods available, but the amount of food consumed should be less than usual.
• Avoid beverages with a high content of sugar; instead drink natural water.
• The child should replace snacks with low-sugar beverages or water and may have a calorie dense snack of his/her choice occasionally (once a week).
Foods to be included in the child's diet:
• Whole grain breads, pasta, rice, cereal.
• Turkey ham, turkey sausages, chicken, tuna, eggs and beans
• Low-fat milk, cheese, yogurt
• Lettuce, tomato, carrots
• Any kind of fruit
Foods to be avoided in the child's diet:
• Foods with a high sugar content such as soft drinks, candy, commercial fruit juices, and chocolate.
• Foods with a high fat content, such as cream, desserts made from whole milk, peanut butter, fried food, pork and lamb meat and their products such as bacon and pork sausage.
Example of a lunch:
• A sandwich with one item (low fat cheese, turkey ham, or tuna fish in water) + 1 fruit or vegetable + natural water or one glass of a low-sugar beverage and the rest of beverages as natural water.

Children in all four groups were evaluated for anthropometry, body composition, physical activity, and blood lipids at the beginning of the study before treatments and after 12 weeks with each respective treatment.

### Anthropometry, body composition and blood lipids

Anthropometric measures included weight and height and were done as described above. Standardization in height and weight measures was done following standard procedures recommended by the World Health Organization [16]. Each child was evaluated by the same observer at basal and post-treatment. Body composition analysis was carried out by bioelectrical impedance using a conductance measurement apparatus (BIA 101, RJL Systems, Clinton TWP, MI). The apparatus was calibrated everyday before measures were carried out. Children were laid down in a bed placed in a quiet room inside the school, apart from where the rest of the measurements took place. Electrodes were placed on the left foot and right hand, after cleaning the area with alcohol. Children were asked to remain calm and not to move for the duration of evaluation.

A fasting blood sample was drawn from every child at basal and after 12 weeks of treatment. Children in all schools were asked to attend at 8 in the morning. Mother and child were instructed that the child should not have any food after 9 p.m. on the night before. Both mother and child were asked before the blood sample was taken if the child had fasted. Blood samples were centrifuged at 1800–2000 rpm during 15 minutes and plasma was stored at -20°C until analysis. Biochemical analysis in plasma samples included triglycerides, total cholesterol and HDL cholesterol and were done using a commercial kit (Sera-Pak Kit Bayer Diagnostics, France).

### Physical activity evaluation

Physical activity of all children was evaluated by asking the child's mother to fill out a questionnaire at the beginning of the study and 12 weeks after treatment began. The questionnaire asked to recall different physical activities normally carried out by children throughout the week as well as their duration. This questionnaire has been validated and applied in previous studies [17]. The outcome of the questionnaires showed the time spent performing different activities during the week. Time of each type of activity was transformed into Metabolic Equivalent units (Mets/hr), which is the ratio of the metabolic rate during the physical activity to the resting metabolic rate, according to the compendium of physical activities from the Prevention Research Center of the University of South Carolina [18]. For data analysis, physical activities were grouped into intense, moderate and low depending on Mets/hr spent as follows: Low = 0 to 3 Mets/hr, Moderate = 3 to 6 Mets/hr and Intense = 6 or more Mets/hr.

### Data analysis

Percent fat and fat free mass were calculated from the reactance and resistance values obtained in the bioimpedance analysis using the equation suggested by Kottler et al. (1996) [19]. LDL and VLDL were calculated from total cholesterol, HDL and triglycerides concentrations [20]. BMI and BMI percentile were calculated in Epi-Info v.3.3.2. Treatment effect was measured as the change on anthropometric and biochemical determinations within initial and final measures and mean change among groups comparison. Partial measurements were analyzed to confirm validity of initial and final measurements. Within effects were carried out with a paired T-Test. Between groups effect in lipids and anthropometry changes was observed with a one-way ANOVA to compare unadjusted changes and with a univariate general linear model adjusted for baseline value, gender and interactions in case they resulted significant and the school random effect. Physical activity analysis was evaluated as the final evaluation controlled for baseline value, gender and the school random effect. Treatments' pairwise comparisons were tested with the least significant difference test [21]. Additionally, an analysis of variance and a chi square test was carried out to compare baseline age, anthropometry and gender of subjects included in the analysis versus children that had missing data and were not included in the analysis. Statistical analyses were performed using the software SPSS, V.9.0 (Chicago, IL).

## Results

**Figure 1:**
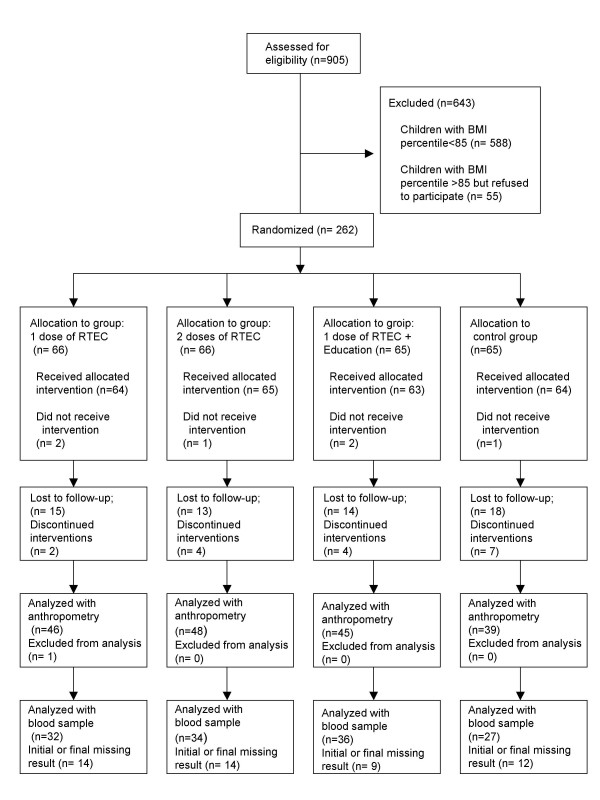
Flow-chart.

Children were recruited from October to December 2002 and the fieldwork was from January to June 2003. The statistical analyses considered all children that had initial and final measurements in an intention to treat basis. Only one child that had an extreme weight final value was excluded from analysis. The participants' flow chart is shown in figure 1. Age, gender and height were not different between children included and children excluded from the analysis. Characteristics of subjects in the experimental groups at the beginning of the study are shown in table 2. Changes in weight, BMI and body composition are shown in Table 3. After 12 weeks of intervention there was a significant increase in body weight in the two RTEC groups and in the control group, only the group that had RTEC plus nutrition education had no increment in body weight. In analysis of variance, children that consumed one serving of RTEC and had nutrition education had a difference in unadjusted weight change of 2.03 kg compared with children in the control group (p < 0.01). Body weight change in the RTEC and nutrition education group adjusted for gender, school and baseline body weight was also significantly different from the control (p < 0.001) and the other two treatment groups (p < 0.01). Unadjusted and adjusted changes in body weight with both treatments with RTEC alone were not statistically different from the control group. BMI reduced significantly only in the group of children that received RTEC and nutrition education (p < 0.01); children in this group had an unadjusted change in mean BMI of 0.64 kg//m^2 ^higher than the control group (p < 0.01). This group's adjusted change in BMI was also statistically greater than control (p < 0.01) and the other two treatments with RTEC only (p < 0.05). Children in the RTEC and nutrition education group showed an unadjusted decrease in total body fat of 1.15 kg compared to the control group (p < 0.05) and the change adjusted for sex, school and baseline body fat was different from the control group and from the group with 1 dose or two of RTEC. Boys reduced total body fat 1.3% more than girls did (p < 0.05) (Data not shown). Unadjusted and adjusted changes in indicators of body composition in the two RTEC groups that did not receive any nutrition education were not different compared with the control group.

**Table 2:** Characteristics of subjects in experimental groups at baseline *†

	1 dose of RTEC	2 doses of RTEC	1 dose of RTEC + Nutrition education guide	Control
N	46	48	45	39
Males %	56.4	40.5	47.5	51.6
Females %	43.6	59.5	52.5	48.4
Age (m)	110.3 ± 19.7	109.3 ± 18.9	107.8 ± 18.8	110.1 ± 18.9
Anthropometry:				
Weight (Kg)	47.0 ± 12.9	47.7 ± 12.7	46.4 ± 12.2	48.2 ± 11.7
Height (Cm)	139.2 ± 12.1	139.01 ± 9.4	138.2 ± 10.8	139.8 ± 11.4
BMI (Kg/M^2^)	23.7 ± 3.3	24.3 ± 3.7	23.8 ± 3.1	24.3 ± 3.1
Height for age (Z-score)	0.4 ± 0.8	0.5 ± 0.8	0.5 ± 0.9	0.6 ± 1.1
Weight for age (Z-score)	2.1 ± 1.0	2.4 ± 1.4	2.2 ± 1.0	2.4 ± 1.0
Weight for height (Z-score)	2.9 ± 1.1	3.0 ± 1.0	2.8 ± 0.7	3.0 ± 0.9
Blood lipids:				
N	27	36	34	32
Total Cholesterol (mg/dL)	141.3 ± 31.3	140.6 ± 32.9	127.4 ± 23.3	138.8 ± 32.9
Triglycerides (mg/dL)	108.6 ± 45.2	132.2 ± 46.4	130.2 ± 47.7	125.1 ± 45.1

* Values are means ± standard deviation unless otherwise is mentioned.

† No statistical significant difference among groups was found

**Table 3:** Effect of treatments on anthropometry and body composition in the different groups *

	1 dose of RTEC	2 doses of RTEC	1 dose of RTEC + Nutrition education guide	Control
N	46	48	45	39
Weight (Kg)				
Initial	47.0 (43.0, 51.1)	47.7 (43.6, 51.8)	47.0 (43.2, 50.8)	48.2 (44.04, 52.3)
Final	47.92 (43.9, 52.0)	48.6 (44.6, 52.7)	46.08 (42.5, 49.7)	49.30 (45.2, 53.4)
Unadjusted change	0.9 (0.4, 1.4) ‡	0.9 (0.3, 1.5) ‡	-0.9 (-2.2, 0.5) §	1.2 (0.8, 1.5) ‡
Adjusted change †	1.03 (0.3, 1.7)	0.6 (-0.1, 1.3)	-1.01 (-1.7, -0.3) **	1.2 (0.4, 2.0)
BMI(Kg/M^2^)				
Initial	23.7 (22.7, 24.8)	24.3 (23.1, 25.5)	24.1 (23.1, 25.2)	24.3 (23.2, 25.4)
Final	23.8 (22.6, 24.9)	24.1 (22.8, 25.3)	23.2 (22.3, 24.1)	24.3 (23.2, 25.4)
Unadjusted change	0.04 (-0.3, 0.4)	-0.2 (-0.5, 0.1)	-1.0 (-1.7, -0.2) ‡, §	0.02 (-0.1, 0.2)
Adjusted change †	0.1 (-0.3, 0.4)	-0.3 (-0.7, 0.1)	-0.9 (-1.2, -0.5) **	0.01 (-0.4, 0.4)
Total Body Fat (%)				
Initial	23.6 (20.6, 26.6)	25.9 (22.8, 28.9)	24.4 (21.6, 27.3)	27.1 (23.9, 30.2)
Final	24.1 (20.9, 27.2)	25.5 (22.5, 28.5)	23.7 (20.7, 26.7)	27.5 (24.5, 30.5)
Unadjusted change	0.5 (-0.1, 1.1)	-0.4 (-1.0, 0.3)	-0.7 (-1.7, 0.3) §	0.4 (-0.4, 1.2)
Adjusted change †	0.4 (-0.4, 1.1)	-0.5 (-1.3, 0.3)	-0.8 (-1.6, -0.04) ††	0.4 (-0.5, 1.4)

* Values are means (95% Confidence Interval).

† Estimated mean change adjusted for initial value, gender, school random effect and significant interactions.

‡Difference between initial and final is significant at p < 0.05 in paired T-Test.

§ Change is different from the control group at p < 0.05 in ANOVA

** Estimated change from group with 1 dose of RTEC + Nutrition education guide is different to all other groups, at p < 0.05 in Adjusted ANOVA

†† Estimated change from group with 1 dose of RTEC + Nutrition education guide is different to group with 1 dose of RTEC and to control group, at p < 0.05 in Adjusted ANOVA

The effect of different treatments on blood lipids is shown in table 4. Only children that had RTEC and nutrition education showed a significant reduction in triglycerides (p < 0.05), an increase in HDL (p < 0.01) and a small reduction in VLDL (p < 0.05). Changes in the other groups were not statistically significant. Comparison of unadjusted changes among groups showed that only HDL increased significantly in the group with RTEC and nutrition education compared to the control group. Treatment changes adjusted for baseline value and school were not different from the control group.

**Table 4:** Effect of treatments on plasma lipids in the different groups *

	1 dose of RTEC	2 doses of RTEC	1 dose of RTEC + Nutrition education guide	Control
N	32	34	36	27
Total Cholesterol				
Initial	143.3 (132.6, 154.1)	141.3 (130.0, 152.7)	128.6 (121.1, 136.1)	134.6 (123.3, 145.8)
Final	149.6 (138.8, 160.3)	147.5 (135.7, 159.4)	136.8 (128.0, 145.6)	141.3 (132.0, 150.7)
Unadjusted change	6.2 (-7.3, 19.7)	6.2 (-8.0, 20.4)	8.2 (-3.4, 19.8)	6.7 (-5.8, 19.3)
Adjusted change †	14.7 (4.5, 24.9)	14.2 (4.3, 24.1)	9.5 (-0.7, 19.6)	6.2 (-4.7, 17.1)
Triglycerides				
Initial	109.5 (92.9, 126.1)	134.2 (118.1, 150.2)	129.5 (113.4, 145.6)	121.9 (106.6, 137.1)
Final	134.5 (109.7, 159.2)	119.4 (102.9, 135.8)	108.7 (92.8, 124.6)	121.6 (102.2, 141.0)
Unadjusted change	25.0 (-3.6, 53.6)	-14.8 (-31.8, 2.2)	-20.7 (-36.4, -5.1) ‡	-0.2 (-19.3, 18.8)
Adjusted change †	13.5 (-6.5, 33.4)	-10.3 (-29.0, 8.4)	-18.1 (-36.7, 0.6)	-4.3 (-24.6, 16.0)
HDL cholesterol				
Initial	48.4 (43.9, 52.8)	48.1 (44.3, 51.8)	43.1 (39.3, 47.0)	47.5 (42.4, 52.6)
Final	47.0 (42.4, 51.6)	48.5 (44.8, 52.2)	49.7 (46.5, 53.0)	44.8 (40.5, 49.1)
Unadjusted change	-1.4 (-7.3, 4.6)	0.4 (-4.6, 5.5)	6.6 (2.2, 11.1) ‡, §	-2.7 (-6.5, 1.1)
Adjusted change †	-2.2 (-5.7, 1.4)	1.0 (-2.4, 4.3)	1.7 (-1.7, 5.1)	-3.0 (-6.7, 0.7)
LDL cholesterol				
Initial	122.6 (111.0, 134.3)	123.6 (112.2, 135.1)	114.1 (104.9, 123.4)	116.7 (106.7, 126.8)
Final	137.1 (123.5, 150.6)	125.2 (112.9, 137.5)	112.3 (101.3, 123.2)	125.4 (114.4, 136.4)
Unadjusted change	14.4 (1.0, 27.8) ‡	1.6 (-11.8, 14.9)	-1.9 (-15.7, 12.0)	8.7 (-5.0, 22.3)
Adjusted change †	19.3 (7.3, 31.3)	9.2 (-2.3, 20.7)	1.8 (-10.0, 13.5)	8.0 (-4.8, 20.8)
VLDL cholesterol				
Initial	21.9 (18.6, 25.2)	26.8 (23.6, 30.0)	25.7 (22.6, 28.9)	24.4 (21.3, 27.4)
Final	26.9 (22.0, 31.9)	23.9 (20.6, 27.2)	21.9 (18.8, 25.1)	24.3 (20.5, 28.2)
Unadjusted change	5.0 (-0.7, 10.7)	-3.0 (-6.4, 0.5)	-3.8 (-6.9, -0.6) ‡	-0.04 (-3.9, 3.8)
Adjusted change †	2.6 (-1.4, 6.6)	-2.0 (-5.8, 1.7)	-3.3 (-7.0, 0.4)	-0.8 (-4.9, 3.2)

* Values are means (95% CI) in mg/dL.

† Estimated mean change adjusted for initial value, gender, school random effect and significant interactions.

‡ Difference between initial and final significant at p < 0.05 in paired T-test.

§ Change in group with 1 dose of RTEC + Nutrition education guide is different to change in group with 1 dose of RTEC and to the control group at p < 0.01 in ANOVA.

Changes ± standard deviation (SD) in intense, moderate and low physical activities in Mets/week were the following: for with 1 dose of RTEC, 13.4 ± 41.3, 4.3 ± 12.7, 3 ± 18, for group with 2 doses of RTEC 2.4 ± 61.5, -2.2 ± 17.1, 2.6 ± 31.9, for with 1 dose of RTEC + education guide -3.6 ± 52.4, -0.2 ± 16, -5 ± 19 and for the control group 4.6 ± 31.6, -1.1 ± 9.9, 6.7 ± 18.5. Changes were not statistically different neither between basal and final evaluations nor among experimental groups. When adjusting model for gender and school, boys increased their intense physical activity while girls decreased it resulting in a significant difference between boys and girls (8.8 ± 60.2 vs 12.6 ± 85.7).

## Discussion

Although there are many environmental factors promoting excess energy intake, consumption of high fat diets increases the likelihood of obesity and the risk of obesity is lower in individuals consuming low fat diets. A review of clinical trials and animal studies [22] suggests that the Public Health recommendation to lower dietary fat intake continues to be an appropriate measure to reduce energy intake and obesity. Fat compared with carbohydrates and proteins, increases the energy density of foods and diets.. Thus, a logical suggestion has been to replace fat with carbohydrate and therefore, decrease the energy density of the diet [5].

The present study showed that the increase in RTEC consumption as a source of carbohydrate in children was effective in reducing body weight and body fat only when a nutrition education guide was included as part of the treatment. The inclusion of either one or two servings of RTEC in the diet without nutrition education was not effective in reducing body fat and did not cause any significant change in body weight compared with the control no-treatment group. Kirk et al [5] found a significant reduction of 2 kg body weight in 29 adults that replaced one meal with a serving of RTEC everyday during 4 weeks as a high carbohydrate regime. Differences between our study and this study include the difference in the population studied but more important is that the study by Kirk et al [5] did not include a control group. This makes its conclusion about the effectiveness of increasing carbohydrate consumption as an effective approach to treat obesity weak. Rodearmel et al. [23] studied the impact of increasing 2 serving of RTEC/day and increasing daily steps in a 13-week intervention study as a family-based approach to prevent obesity and found significant differences in children's BMI-for-age and body fat between the experimental and the control groups. The control group in this study did not receive any intervention, therefore, the effect of the RTEC seen cannot be separated from the increase in physical activity in the children.

Our study agrees with other studies [5,23] in the fact that an increase in RTEC consumption as a source of carbohydrates was shown to be an effective strategy to lose weight in obese children but our study suggests that only when it is given with nutrition education. The change in body weight in the group receiving education and RTEC was accompanied with a reduction in total body fat. These changes did not occur in the groups that received one or two servings of RTEC and that the mother and child did not receive any nutrition education guide. These findings suggest that in our population a nutrition education guideline might be necessary for the beneficial effects of increasing carbohydrate consumption.

The importance of education programs in the treatment of obesity has been known for a number of years, but only recently it has been suggested that nutrition education should be part of any successful strategy to reduce obesity in children [24-29], adolescents [27-30] and in adults [31,32]. Also, nutrition education has proven to be effective in improving nutritional status of individuals in different populations at risk. Studies of nutrition education programs that are continuous, specific, focused and targeted to vulnerable populations have been successful in improving nutritional status [33-37]. Our study suggests that providing a nutrition education guide makes dietary changes, such as increase in carbohydrate consumption, more effective, and that a lack of an adequate nutritional education can cause nutritional strategies to fail.

It is important to consider that since we did not include a group receiving nutrition education alone, we are unable to conclude that the group receiving RTEC in addition to a nutrition education program works any better than nutrition education alone to increase carbohydrate intake. The study was not designed to test the effect of a nutrition education program without the increase in RTEC consumption.

The reduction in body fat and body weight in the RTEC and nutrition education group of the children was accompanied by a significant reduction in plasma triglycerides and by an elevation in HDL. Changes in these two variables are clearly associated with a reduction in body fat and are beneficial to reduce health complications associated with excess of body fat.

## Conclusion

We found that a strategy to increase carbohydrate consumption to reduce obesity in children is effective only when accompanied with a nutrition education program. An increase in RTEC intake as a source of carbohydrates with a simple nutrition education guideline produced a significant loss of body weight, a decrease in body fat and in plasma triglycerides, and an increase in high density lipoproteins. The importance of nutrition education could be extrapolated to other nutritional manipulations intended for treatment of obesity.

## Consent

Written informed consent was obtained from the parents for participation and publication of results. A copy of the written consent is available for review by the Editor-in-Chief of this journal. This study was approved by the Internal Committee of Human Research of the University of Queretaro.

## Competing interests

The authors declare that they have no competing interests.

## Authors' contributions

JLR developed the study design, supervised the study, and made a substantial contribution with interpretation of data, drafting the manuscript and revising it critically for important intellectual content. MRA participated in the study design and coordinated the field research. KM coordinated the field research and supervised the quality of data collection. OPG contributed with the study conception and design, interpretation of data and writing the publication. And MCC participated in managing the data, carried out the statistical analysis and contributed to revising the manuscript. All authors read and approved the final manuscript.
